# Patients with Community Acquired Pneumonia Exhibit Depleted Vitamin C Status and Elevated Oxidative Stress

**DOI:** 10.3390/nu12051318

**Published:** 2020-05-06

**Authors:** Anitra C. Carr, Emma Spencer, Liane Dixon, Stephen T. Chambers

**Affiliations:** 1 Nutrition in Medicine Research Group, Department of Pathology and Biomedical Science, University of Otago, Christchurch 8011, New Zealand; emma.spencer@otago.ac.nz; 2 Department of Infectious Diseases, Christchurch Hospital, Christchurch 8011, New Zealand; liane.dixon@cdhb.health.nz; 3 The Infection Group, Department of Pathology and Biomedical Science, University of Otago, Christchurch 8011, New Zealand; steve.chambers@otago.ac.nz

**Keywords:** vitamin C, ascorbic acid, ascorbate, pneumonia, community acquired pneumonia, oxidative stress, protein carbonyls, hypovitaminosis C, vitamin C deficiency

## Abstract

Pneumonia is a severe lower respiratory tract infection that is a common complication and a major cause of mortality of the vitamin C-deficiency disease scurvy. This suggests an important link between vitamin C status and lower respiratory tract infections. Due to the paucity of information on the vitamin C status of patients with pneumonia, we assessed the vitamin C status of 50 patients with community-acquired pneumonia and compared these with 50 healthy community controls. The pneumonia cohort comprised 44 patients recruited through the Acute Medical Assessment Unit (AMAU) and 6 patients recruited through the Intensive Care Unit (ICU); mean age 68 ± 17 years, 54% male. Clinical, microbiological and hematological parameters were recorded. Blood samples were tested for vitamin C status using HPLC with electrochemical detection and protein carbonyl concentrations, an established marker of oxidative stress, using ELISA. Patients with pneumonia had depleted vitamin C status compared with healthy controls (23 ± 14 µmol/L vs. 56 ± 24 µmol/L, *p* < 0.001). The more severe patients in the ICU had significantly lower vitamin C status than those recruited through AMAU (11 ± 3 µmol/L vs. 24 ± 14 µmol/L, *p* = 0.02). The pneumonia cohort comprised 62% with hypovitaminosis C and 22% with deficiency, compared with only 8% hypovitaminosis C and no cases of deficiency in the healthy controls. The pneumonia cohort also exhibited significantly elevated protein carbonyl concentrations compared with the healthy controls (*p* < 0.001), indicating enhanced oxidative stress in the patients. We were able to collect subsequent samples from 28% of the cohort (mean 2.7 ± 1.7 days; range 1–7 days). These showed no significant differences in vitamin C status or protein carbonyl concentrations compared with baseline values (*p* = 0.6). Overall, the depleted vitamin C status and elevated oxidative stress observed in the patients with pneumonia indicates an enhanced requirement for the vitamin during their illness. Therefore, these patients would likely benefit from additional vitamin C supplementation to restore their blood and tissue levels to optimal. This may decrease excessive oxidative stress and aid in their recovery.

## 1. Introduction

Pneumonia is a severe lower respiratory tract infection that can be caused by bacterial, fungal and viral pathogens, including the novel severe acute respiratory syndrome coronavirus (SARS-CoV-2) [1,2]. Lower respiratory tract infections are the leading cause of morbidity and mortality for communicable disease worldwide [3]. In 2016, lower respiratory tract infections resulted in more than 65 million hospital admissions and nearly 2.4 million deaths worldwide [4]. Mortality is particularly high for children under five and the elderly, which is of concern due to the increasingly aging population [5]. Increased incidence of community-acquired pneumonia is also associated with lower socioeconomic status and certain ethnic groups [5,6,7].

Pneumonia is a common complication and a major cause of mortality of the vitamin C deficiency disease scurvy, which suggests an important link between vitamin C status and lower respiratory tract infections [8]. The EPIC-Norfolk longitudinal study comprising more than 19,000 men and women has shown a 30% lower risk of pneumonia and a 39% lower mortality from pneumonia for people in the top quartile of vitamin C status (>66 µmol/L, i.e., saturating status), ascertained from a single baseline measurement at enrolment, compared with those in the bottom quartile of vitamin C status (≤41 µmol/L) [9]. Despite the known roles of vitamin C in supporting immune function through acting as an antioxidant and enzyme cofactor [10], surprisingly few studies have explored the link between vitamin C and pneumonia [11]. Two case control studies have indicated that patients with pneumonia have significantly lower vitamin C status than healthy controls, and there was an inverse correlation with the severity of the condition [12,13]. Two other studies that explored the time course indicated that up to 40% of patients with pneumonia exhibited vitamin C deficiency (i.e., plasma vitamin C concentrations <11 μmol/L) at hospital admission, and concentrations remained low for at least four weeks [14,15]. 

These studies indicate a higher utilization of, and potentially also a higher requirement for, vitamin C during lower respiratory tract infections. Patients with pneumonia may also have lower baseline vitamin C status, which could potentially make them more susceptible to infection. Due to the paucity of recent studies investigating the link between vitamin C and pneumonia, and the global relevance due to periodic outbreaks of SARS-related coronaviruses, we measured the vitamin C status of patients with community-acquired pneumonia who were admitted to the Acute Medical Assessment Unit or Intensive Care Unit of our public hospital. We also measured protein carbonyl concentrations, an established marker of oxidative stress. These parameters were compared with those measured in healthy controls. 

## 2. Methods 

### 2.1. Setting and Study Participants

Christchurch Hospital is the largest tertiary, teaching and research hospital in the South Island of New Zealand; it is located in New Zealand’s second largest city and services 600,000 people in the Canterbury region. A total of 50 patients were recruited for this observational study in Christchurch Hospital; 44 patients with community-acquired pneumonia were recruited in the Acute Medical Assessment Unit and medical wards (July 2017 to February 2018) and six patients with CAP were recruited in the Intensive Care Unit (December 2015 to August 2016). Ethical approval was obtained from the New Zealand Southern Health and Disability Ethics Committee (#16STH235 and #15STH36). Radiology reports for suspected pneumonia were viewed daily to identify potential patients. Many of the patients were elderly and to ensure a correct consent process, cognition was considered, along with a supportive family, to discuss this before continuing. All patients signed informed consent documents. 

### 2.2. Inclusion and Exclusion Criteria

Community-acquired pneumonia was defined as a pneumonia that had been acquired outside of hospitals or health care settings. Pneumonia was defined in a patient with an acute illness and new inflammatory infiltrate on a chest radiograph, or a diagnosis of community-acquired pneumonia by the treating physician and the presence of at least one of the following acute respiratory signs and symptoms: Cough, increased sputum production, dyspnoea, core body temperature of ≥38.0 °C and auscultatory findings of abnormal breathing sounds or rales [16,17]. Other inclusion criteria were age ≥ 18 years and the ability to provide informed consent. Exclusion criteria were as follows: (1) pneumonia was (a) not the primary cause for hospital admission, (b) an expected terminal event or (c) distal to bronchial obstruction; (2) patients with tuberculosis or bronchiectasis; and (3) patients who had been in hospital within the previous 14 days, or had previously been entered in the study. 

### 2.3. Disease Severity Scores

The CURB-65 score (range 0–5) was calculated from admission records using values from the first 12 h in hospital to determine severity and predict mortality of pneumonia and was calculated for all patients. The criteria were as follows: confusion of new onset (defined as an abbreviated mental test score (AMTS) of ≤8), blood urea nitrogen > 7 mmol/L, respiratory rate ≥ 30 breaths per minute, blood pressure < 90 mmHg systolic or ≤60 mmHg diastolic and age ≥ 65 years. Each criteria scored 1 point if met [18]. ICU severity scores were recorded if the patients were admitted to the ICU. These were Acute Physiology and Chronic Health Evaluation II and III score (APACHE II and III, range 0–79 and 0–299, respectively); Simplified Acute Physiology Score II (SAPS II, range 0–163); and Sequential Organ Failure Assessment score (SOFA, range 0–24). Common comorbidities were also recorded.

### 2.4. Blood Sampling and Processing

A blood sample (with heparin anticoagulant) was collected within 24 h of admission and pneumonia was confirmed via chest film. Because these were acutely ill patients, the blood samples were non-fasting. A blood sample was not able to be collected from one participant so their clinical data were excluded from the analysis. A second sample was collected from a subset of the participants on day of discharge (*n* = 14). Blood samples were also collected from a cohort of non-fasting healthy community controls who were resident in Christchurch (*n* = 50, 50% female, aged 57 ± 17 years). The blood samples were placed on ice and immediately transferred to the laboratory for centrifugation to separate plasma for vitamin C and oxidative stress biomarker analysis. An aliquot of the plasma was treated with an equal volume of 0.54 M perchloric acid and 100 µmol/L of the metal chelator DTPA to precipitate protein and stabilize the vitamin C. The supernatant and spare plasma samples were stored at −80 °C until analysis.

### 2.5. Analysis of Blood Analytes

Routine hematological analyses were carried out at Canterbury Health Laboratories, an International Accreditation New Zealand (IANZ) laboratory. Identified organisms were recorded. The vitamin C content of the processed plasma samples was determined using HPLC with electrochemical detection, as described previously [19]. The protein carbonyl content of the plasma was determined using a sensitive ELISA method, as described previously [20]. 

### 2.6. Statistical Analysis

Data are presented as mean and SD or mean and 95% CI as indicated. Statistical analyses were carried out using Excel data analysis add-in and GraphPad Prism 8.0 software (San Diego, CA, USA). Differences between groups were determined using Student’s *t*-test or Mann–Whitney U test for non-parametric variables. Linear regression analyses were carried out using Pearson correlations. Statistical significance was set at *p* < 0.05.

## 3. Results

### 3.1. Participant Characteristics

Participants were recruited in the Acute Medical Assessment Unit (AMAU; *n* = 44) and the Intensive Care Unit (ICU; *n* = 6). One of the AMAU patients was transferred to the ICU (for 12 days) following baseline measurements. There were 54% males in the cohort and a mean age of 68 ± 17 years (Table 1). The most common comorbidities were cardiovascular diseases, asthma and chronic heart failure. Mean CURB-65 score for the cohort was 1.9: 1.8 for the AMAU patients and 3.5 for the ICU patients (excluding two ICU patients who had transferred from other hospitals and so had lower CURB-65 scores at admission). Mean length of hospital stay (LOS) was 3 days, with a higher mean LOS of 24 days for the ICU cohort. Two of the patients in the AMAU cohort died (of cardiac complications) giving an overall mortality of 4% for the total cohort. 

The ICU cohort exhibited more severe hematological parameters, including a significantly higher mean C-reactive protein concentration (*p* = 0.027; Table 2). *Streptococcus pneumonia* was the most common pathogen identified in the AMAU patients (11%), followed by *Legionella* (9%) and *Haemophilus influenzae* (7%), and 11% of the patients had viral pathogens identified (*Adenovirus, Influenza A, Parainfluenza* and *Rhinovirus).* Although *Pseudomonas aeruginosa* was the most common pathogen identified in the ICU patients, the role in causing the initial pneumonia was unclear.

### 3.2. Vitamin C and Protein Carbonyls

**Figure 1:**
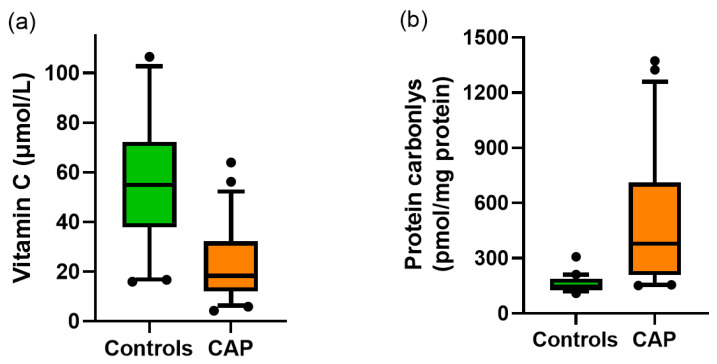
Vitamin C status and protein carbonyl concentrations in the community-acquired pneumonia (CAP) cohort and healthy controls. (**a**) Vitamin C was measured using HPLC with electrochemical detection, *p* < 0.001, *n* = 50 for both controls and CAP. (**b**) Protein carbonyls were measured using ELISA; *p* < 0.001, *n* = 50 for controls, *n* = 46 for CAP. Box plots show median with 25th and 75th percentiles as boundaries, and whiskers are the 5th and 95th percentiles, with symbols indicating outlying data points.

The vitamin C status of community-acquired pneumonia patients admitted to hospital was 23 ± 14 µmol/L; this was significantly lower than healthy controls (56 ± 24 µmol/L; *p* < 0.001; Figure 1a). The patients admitted to the ICU had significantly lower vitamin C status than those admitted to AMAU (11 ± 3 µmol/L vs. 24 ± 14 µmol/L; *p* = 0.02). Protein carbonyls, an established biomarker of oxidative stress, were significantly elevated in patients with pneumonia compared with healthy controls (468 ± 305 vs. 159 ± 39 pmol/mg protein, respectively; *p* < 0.001; Figure 1b). 

**Figure 2:**
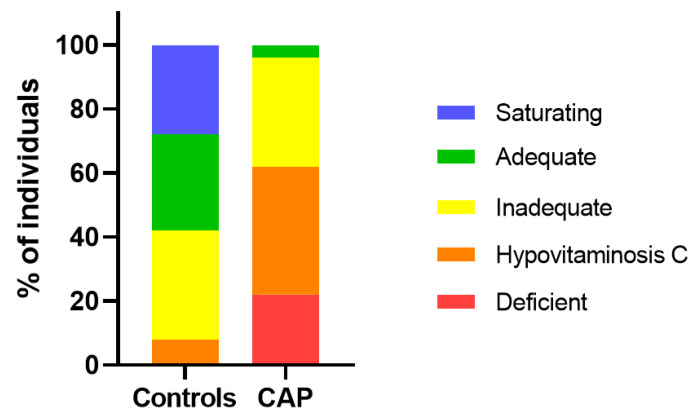
Percentage of individuals from the community-acquired pneumonia (CAP) cohort and healthy controls in different vitamin C categories. Vitamin C categories are presented as saturating (>70 µmol/L), adequate (between 70 and 50 µmol/L), inadequate (between 50 and 23 µmol/L), hypovitaminosis C (between 23 and 11 µmol/L), deficient (<11 µmol/L).

The pneumonia cohort comprised 96% patients with inadequate vitamin C status (i.e., <50 µmol/L), 62% with hypovitaminosis C (i.e., <23 µmol/L) and 22% with frank deficiency (i.e., <11 µmol/L; Figure 2). In contrast, the healthy controls comprised only 8% with hypovitaminosis C and no cases of deficiency.

Subsequent samples were collected from 14 (28%) of the participants (mean 2.7 ± 1.7 days, range 1–7 days); there were no statistically significant differences between baseline and subsequent samples for either vitamin C status or protein carbonyl concentrations (Table 3). 

### 3.3. Biomarker Correlations with Clinical and Physiological Parameters

There was no significant correlation between vitamin C status and CURB-65 scores (*p* = 0.3); however, there was a significant correlation with systolic blood pressure (R = 0.33, *p* = 0.02) and a trend towards significance with diastolic blood pressure (R = 0.26, *p* = 0.07). There was no significant correlation between vitamin C status and C-reactive protein concentrations (R = 0.25, *p* = 0.09). In contrast, there was a significant correlation between protein carbonyls and the CURB-65 score (R = 0.4, *p* = 0.006), as well as with urea and creatinine (R = 0.50–0.58, *p* < 0.001). There was no correlation between protein carbonyls and vitamin C status in the pneumonia cohort (*p* = 0.3); however, an inverse correlation between protein carbonyls and vitamin C status was observed in the healthy controls (R = −0.38, *p* = 0.006). 

## 4. Discussion

Our study shows that patients with community-acquired pneumonia have significantly depleted vitamin C status (mean of 23 µmol/L) and a high prevalence of hypovitaminosis C and deficiency (62% and 22%, respectively). Lower vitamin C status was evident with increasing severity of the condition, as shown by the ICU patients having significantly lower vitamin C status than the rest of the hospitalized cohort. Our data is in agreement with two early observational studies investigating vitamin C status in patients with pneumonia [12,13]. Vitamin C concentrations of 31 µmol/L were reported in 11 patients with pneumonia, compared with concentrations of 66 µmol/L in 20 healthy controls [12]. In a very early study from the 1950s, ascorbate concentrations of 17 µmol/L in 7 acute patients who died, 24 µmol/L in 15 acute survivors, and 34 µmol/L in 13 convalescent cases were compared with 49 µmol/L in 28 healthy controls [13]. Thus, this study supports our finding of lower vitamin C status in more severe cases. These investigators also reported elevated concentrations of oxidized vitamin C (dehydroascorbic acid) in the patient samples, suggesting enhanced oxidative stress; however, the high levels reported in this study are likely an ex vivo artifact of the assay method used [21].

We were able to collect subsequent samples from a subgroup of the patients, which showed no change in vitamin C status in samples collected up to a week later. This is in agreement with an earlier intervention study by Hunt et al., who reported baseline vitamin C levels of 23 µmol/L in 57 elderly people with pneumonia or bronchitis, while two weeks later levels remained in the hypovitaminosis C range (i.e., 19 µmol/L) in the participants who did not receive vitamin C (n = 29) [14]. Another study also showed hypovitaminosis C in 70 patients with acute pneumonia at days 5 and 10 [15]. By week four, both of these studies reported that vitamin C status was still low and only approaching baseline levels [14,15]. This indicates that the vitamin C status of patients with pneumonia is likely to remain low for a significant duration following their illness.

In our study we measured protein carbonyls as a marker of oxidative stress. Protein carbonyls can be formed by a variety of reactive oxygen species and via a number of different reaction pathways including direct oxidation of specific amino acids, oxidative cleavage of the protein backbone or reaction of reactive sugar- and lipid-derived aldehydes with specific amino acids [22]. We found significantly elevated protein carbonyls in the patients with pneumonia compared with healthy controls. Elevated protein carbonyls have been observed previously in critically ill patients with sepsis [23]. The patients with the highest protein carbonyl values measured in our study had hypovitaminosis C. Elevated oxidative stress could be both a cause and a consequence of the low vitamin C status observed in the patients. Interestingly, a pre-clinical animal model indicated that administration of high-dose vitamin C to mice with sepsis decreased protein carbonyl levels in the most severely ill [24]. This has not yet been demonstrated in human studies; however, based on the potent antioxidant properties of vitamin C, it is possible that vitamin C administration to patients with pneumonia could decrease markers of excessive oxidative stress.

Vitamin C is known to have numerous immune supporting functions, including enhancing various leukocyte functions such as chemotaxis and microbial killing (reviewed in [10]). It may also be able to support resolution of the inflammatory process by enhancing neutrophil apoptosis and clearance from the lungs [25,26], as well as limiting generation of neutrophil extracellular traps (NETs), which are thought to enhance tissue damage and prolong lung inflammation [27,28]. Although we did not measure the vitamin C content of leukocytes, Hunt et al. have shown that the vitamin C content of leukocytes is low in patients with acute respiratory infections and these remain low at week 2, although some recovery is observed by week 4 [14]. It is possible that these depleted vitamin C levels may affect neutrophil function [10]. The vitamin C content of neutrophils has also been measured during upper respiratory tract infections such as the common cold [29,30]. Although vitamin C levels do decrease in leukocytes during the common cold, the levels recover more rapidly (i.e., within 4–5 days) than was observed for leukocytes from pneumonia patients. This indicates a prolonged burden on the immune cells of patients with pneumonia, which may affect their recovery.

Surprisingly few vitamin C intervention studies have been carried out in patients with pneumonia [31,32]. Administration of vitamin C at a dose of 200 mg/d to 28 elderly patients with pneumonia and bronchitis restored saturating vitamin C status within two weeks and also improved leukocyte vitamin C concentrations [14]. This study also showed a decreased respiratory symptom score in the most severely ill and a trend towards decreased mortality. Another intervention study showed a dose dependent decrease in duration of hospital stay from 24 days in the control group to 19 days in the lower vitamin C group (250–800 mg/d) and 15 days in the higher vitamin C group (500–1600 mg/d) [15]. Thus, administration of vitamin C to depleted pneumonia patients may improve their clinical outcomes. More well-controlled trials are required to test this [32].

Sepsis is a common complication of severe pneumonia, often requiring admission to the ICU. We have previously shown that critically ill patients with sepsis have depleted vitamin C status despite receiving recommended enteral and parenteral intakes of vitamin C (up to a mean of 200 mg/d) [33]. Other clinical research has indicated that critically ill patients likely need at least 10-fold more vitamin C (i.e., 2–3 g/d) to compensate for the enhanced requirements for the vitamin during the inflammatory process [34,35]. Therefore, it is likely that pneumonia patients who have progressed to sepsis and have been admitted to the ICU will require gram intravenous (IV) doses of vitamin C to restore adequate vitamin C status. One trial has shown improved radiologic score and decreased mortality in severe pneumonia cases in the ICU who were administered a combination containing IV vitamin C at a dose of 6 g/d [36]. A number of other clinical trials of patients with sepsis and septic shock have also indicated improved patient outcomes with administration of gram doses of IV vitamin C [11]. The decreased requirement for vasopressor drugs observed in some trials may reflect the ability of vitamin C to aid in endogenous vasopressor synthesis via its enzyme cofactor functions [37]. In support of this premise, we observed a positive correlation between vitamin C status and blood pressure in the patients with pneumonia.

Although the samples in our study were collected prior to the SARS-CoV-2 outbreak, it is likely that people with COVID-19-associated pneumonia and sepsis would have similarly low vitamin C status and high oxidative stress. Early case reports from the 1940s indicated that IV administration of gram doses of vitamin C to cases of viral pneumonia rapidly improved common symptoms [38]. There are currently a number of intervention trials up and running around the world that will specifically test IV vitamin C for COVID-19-related pneumonia and sepsis. Furthermore, it is likely that patients with other severe infectious conditions may also have low vitamin C status. This has been previously demonstrated in patients with tuberculosis, bacterial meningitis, tetanus and typhoid fever [12,13]. These patients would also likely benefit from additional vitamin C supplementation.

A limitation of our study was the use of non-fasting plasma samples for analysis of vitamin C. However, as these were severely ill hospitalized patients it was felt that fasting was not appropriate. Therefore, we collected non-fasting samples from the healthy controls for an equivalent comparison. The number of participants was relatively low, particularly for the ICU subgroup comparisons; however, the differences in biomarker values between the patients and healthy controls were still highly significant.

## 5. Conclusions

Patients with pneumonia exhibit low vitamin C status and an elevated prevalence of hypovitaminosis C and deficiency compared with healthy controls. This indicates an enhanced requirement for the vitamin during their illness. Due to the important roles that vitamin C plays in the immune system, low vitamin C status is possibly both a cause and a consequence of the disease. The patients also exhibited elevated oxidative stress as evidenced by significantly higher protein carbonyl concentrations than healthy controls. Elevated oxidative stress could be both a cause and a consequence of the low vitamin C status observed in the patients. Therefore, these patients would likely benefit from additional vitamin C supplementation to restore their blood and tissue levels to optimal. This may decrease excessive oxidative stress and aid in their recovery.

## Figures and Tables

**Table 1:** Participant baseline characteristics.

Characteristic	Total Cohort (*n* = 50)	AMAU (*n* = 44)	ICU (*n* = 6)
Age, years ^1^	68 (17)	66 (22)	58 (17)
Male sex, n (%)	27 (54)	23 (52)	4 (67)
Comorbidities, n (%)		COPD 6 (14) Asthma 11 (25) Chronic heart failure 9 (20) Cardiovascular disease 19 (43) Diabetes 5 (11) Cerebrovascular disease 6 (14) Renal disease 3 (7) Solid organ malignancy 2 (5) Hematological malignancy 1 (2) Immune suppression 2 (5)	COPD 1 (17) Cardiovascular disease 1 (17) Renal failure 1 (17) Hematological malignancy 1 (17) Immune compromised 1 (17)
Temperature, °C		38 (1.2)	
Hypothermia, n (%)		2 (4.5)	
Systolic BP	139 (38)	147 (33)	80 (11)
Diastolic BP	75 (20)	79 (18)	46 (10)
Heart rate, beats/min		106 (22)	
Respiratory rate, breaths/min			36 (11)
Radiological confirmation, n (%)		37 (84)	
CURB-65 score (0–5)	1.9 (1.3)	1.8 (1.3)	3.5 (0.6) ^2^
SAPS II score (0–163)			40 (11)
APACHE II score (0–79)			20 (6)
APACHE III score (0–299)			72 (24)
SOFA score (0–24)			10 (3)
Vasopressors, n (%)		0	6 (100)
Mechanical ventilation, n (%)		0	4 (67)
FiO_2_			0.38 (0.07)
Hospital LOS, days ^3^	3 (0–99)	3 (0–91)	24 (5–99)
Mortality, n (%)	2 (4.0)	2 (4.5)	0

^1^ Data is presented as mean (SD) unless otherwise indicated; ^2^ Two of the ICU patients were transferred from other hospitals and were excluded from CURB-65 calculations; ^3^ Data is presented as median and range. Key: AMAU, Acute Medical Assessment Unit; APACHE, Acute Physiology and Chronic Health Evaluation; COPD, chronic obstructive pulmonary disease; CURB, confusion urea respiratory rate blood pressure; ICU, intensive care unit; LOS, length of stay; SAPS, simplified acute physiology score; SOFA, sequential organ failure assessment.

**Table 2:** Participant hematological parameters.

Parameter	Total Cohort (*n* = 50)	AMAU (*n* = 44)	ICU (*n* = 6)
White cell count (×10^9^/L) ^1^	13 (6)	13 (5)	18 (13)
Neutrophils (×10^9^/L)	11 (5)	11 (5)	15 (10)
Hemoglobin (g/L)	130 (17)	130 (17)	125 (26)
Platelets (×10^9^/L)	241 (117)	244 (91)	218 (227)
Urea (mmol/L)	8.4 (6.5)	7.4 (4.1)	15 (13)
Creatinine (µmol/L)	107 (61)	98 (29)	168 (138)
Bilirubin (µmol/L)	19 (11)	18 (9)	25 (13)
C-reactive protein (mg/L)	165 (127)	152 (127)	261 (78) ^2^
Alanine transaminase (U/L)	34 (21)	33 (21)	35 (23)
Alkaline phosphatase (U/L)		112 (53)	
Lactate (mmol/L)			1.5 (0.8)
PaO_2_ (mmHg)			79 (19)
PaO_2_/FiO_2_			212 (59)

^1^ Data is presented as mean (SD), ^2^ *p* = 0.027 relative to AMAU value. Key: AMAU, Acute Medical Assessment Unit; ICU, intensive care unit.

**Table 3:** Vitamin C status and protein carbonyl concentrations measured over time.

Biomarker	Baseline Sample ^1^	Second Sample ^2^	*p* Value
Vitamin C (µmol/L)	20 (7)	22 (7)	0.6
Protein carbonyls (pmol/mg protein)	541 (157)	625 (252)	0.6

^1^ Data expressed as mean (95% CI); ^2^ Mean 2.7 ± 1.7 days (range 1–7 days).
